# Diet rich in Docosahexaenoic Acid/Eicosapentaenoic Acid robustly ameliorates hepatic steatosis and insulin resistance in seipin deficient lipodystrophy mice

**DOI:** 10.1186/s12986-015-0054-x

**Published:** 2015-12-18

**Authors:** Pengfei Xu, Huan Wang, Abudurexiti Kayoumu, Mengyu Wang, Wei Huang, George Liu

**Affiliations:** Institute of Cardiovascular Sciences, Key Laboratory of Molecular Cardiovascular Sciences, School of Basic Medical Sciences, Peking University Health Science Center, 38, XueYuan Road, HaiDian District, Beijing, 100191 People’s Republic of China

**Keywords:** n-3 polyunsaturated fatty acids, Eicosapentaenoic acid (EPA), Docosahexaenoic acid (DHA), Hepatic steatosis, Insulin resistance, De novo lipogenesis, β-oxidation, Seipin, Congenital generalized lipodystrophy

## Abstract

**Background:**

N-3 polyunsaturated fatty acids (n-3 PUFAs), in particular eicosapentaenoic acid (EPA) and docosahexaenoic acid (DHA), have been shown to effectively improve hepatic steatosis and insulin resistance caused by obesity. Lipodystrophy could also develop insulin resistance and hepatic steatosis. However, the effect of supplemental DHA/EPA to hepatic steatosis caused by lipodystrophy is unknown. In this study, we investigated whether a diet rich in n-3 PUFAs could ameliorate severe steatosis in lipoatrophic seipin gene knockout (SKO) mice.

**Methods:**

Eight-week-old C57BL/6 J WT and SKO mice were fed with normal chow diet (NC), or 2 % DHA/EPA (3:1) diet for 12 weeks. Total cholesterol (TC) and triglycerides (TG) in plasma and liver, plasma high density lipoprotein-cholesterol (HDL-C), glucose (Glu), insulin, leptin and adiponectin levels were measured. Gene regulations and protein levels were investigated using quantitative PCR and western blot in liver.

**Results:**

We found that the DHA/EPA diet protected against hepatic steatosis effectively in SKO mice morphologically. Hepatic TG content was decreased about 40 % (*p* < 0.05) in SKO mice fed with the DHA/EPA diet compared to chow fed SKO controls. Glucose and insulin tolerance were also improved significantly in SKO mice with DHA/EPA diet.

In analyzing hepatic gene expression pattern it was found that TG synthesis related genes, such as carbohydrate response element binding protein (ChREBP), stearoyl-CoA desaturase 1 (SCD1) and fatty acid synthase (Fas) were upregulated in SKO mice compared to WT mice but were significantly decreased in SKO mice on DHA/EPA diet. Fatty acid β-oxidation related genes, on the other hand, such as peroxisome proliferator-activated receptor α (PPARα), carnitine palmitoyltransferase (CPT) and acyl-CoA oxidase 1 (ACOX1) were elevated in both WT and SKO groups on DHA/EPA diets. The protein levels of PPARα, SCD1, CPT1α, Insulin receptor substrate 1 (IRS1) and ratio of p-AKT to AKT showed the same tendency as the result of genes expressions.

**Conclusions:**

The results suggest that n-3 PUFAs rich diet ameliorates lipodystrophy-induced hepatic steatosis through reducing TG synthesis, improving insulin resistance and enhancing β-oxidation in SKO mice.

## Background

Non-alcoholic fatty liver disease (NAFLD) has been recognized as a major health burden and as the most important cause of chronic liver disease [1, 2]. NAFLD is characterized by hepatocyte triacylglycerol accumulation (steatosis), which can progress to inflammation, fibrosis, and cirrhosis. Notably, NAFLD is strongly associated with insulin resistance, type 2 diabetes and obesity [1–3].

Omega-3 polyunsaturated fatty acids (n-3 PUFAs), in particular eicosapentaenoic acid (EPA) and docosahexaenoic acid (DHA), have been shown to ameliorate hepatic steatosis in obese humans and rodents with high fat diet (HFD). It could decrease the endogenous lipid production by decreasing gene expressions of sterol regulatory element binding protein 1c (SREBP1c) and carbohydrate responsive element-binding protein (ChREBP), which were activated in NAFLD [4, 5]. Dietary intake of n-3 PUFAs could improve insulin tolerance in obese mice. N-3 PUFAs could increase expressions of genes involved in insulin sensitivity: peroxisome proliferator activated receptor-γ (PPARγ), glucose transporter (GLUT-2/GLUT-4), and insulin receptor substrates (IRS1/IRS2) were upregulated by n-3 PUFAs in obese mice [6]. Diet with n-3 PUFAs also stimulated hepatic β-oxidation through upregulated expression of peroxisome proliferator activated receptor-α (PPARα) and carnitine palmitoyltransferase (CPT) [7, 8].

NAFLD could not only be induced by obesity, but could also be caused by lipodystrophy, including congenital generalized lipodystrophy (CGL) and acquired lipodystrophy related to HIV-infected patients with antiretroviral treatment [9, 10]. CGL, also known as Berardinelli-Seip congenital lipodystrophy (BSCL), is a rare autosomal recessive disorder characterized by lack of body fat since birth, which results in striking muscular appearance. Patients develop extreme hepatic steatosis, hyperlipidemia and insulin resistance [11]. The BSCL2, seipin gene mutation exhibits the most severe phenotype of lipodystrophy among the CGLs in human [12]. There were also severe insulin resistance and hepatic steatosis in the seipin gene knockout (SKO) mice constructed by our group, however, no hypertriglyceridemia (HTG) was detected [13]. It demonstrates that increased de novo lipogenesis and reduced β-oxidation in liver contribute to hepatic steatosis in SKO mice [14]. However, the effect of supplemental DHA/EPA to hepatic steatosis caused by lipodystrophy is unknown. In this study, we investigated whether a diet rich in n-3 PUFAs could ameliorate severe steatosis in lipoatrophic seipin gene knockout mice.

DHA/EPA are the major components of n-3 PUFAs. Comparing the efficacy of EPA with DHA to hepatic steatosis, both EPA and DHA equally lowered hepatic lipid content, but the efficacy of dietary DHA suppressing hepatic markers of inflammation, fibrosis, and oxidative stress is significantly greater than dietary EPA [15]. DHA could reduce insulin resistance which may be mediated through an increase in circulating adiponectin [16]. But the effect of n-3 PUFAs in lowering plasma triglycerides (TG) is well established mainly in EPA [17, 18]. In this study, we adjusted contents of the mixture to those of tuna oil: 23 % DHA and 7 % EPA (DHA/EPA = 3:1) as previous described [19].

Supplemental n-3 PUFAs have an ameliorative effect in reducing hepatic lipid content in NAFLD caused by obesity, but the effects on NAFLD induced by lipodystrophy are still unknown. In this study, we intended to assess the effects of DHA/EPA on the NAFLD caused by CGL and explore its mechanisms of the treatment.

## Methods

### Animals and diets

Homozygous seipin gene knockout (SKO) mice and littermate wild type (WT) mice with C57BL/6 background were obtained and reproduced as described previously [13]. The SKO and the WT mice with age of 8-week-old were randomly assigned in two groups (9 male mice and 9 female mice for each group), fed ad libitum with normal chow diet (NC) containing 4 % fat by weight, or supplemented with 2 % n-3 PUFAs (20 g/kg; a great gift from Abbott), constituted by DHA/EPA (D/E, 3:1). Food consumptions were measured every 2 weeks during experiment. At 3 months after the treatment,the animals were anesthetized with 1 % pentobarbital sodium (45 mg/kg by i.p.) and blood samples were taken from the retro-orbital plexus at 4 h after fasting. The total fat including subcutaneous, inguinal, gonadal, retroperitoneal and mesenteric fat were dissected and weighted as described previously [13]. The liver was then harvested, weighed and then stored at −80 °C for real-time PCR and western blot analysis. All experiments involving mice were approved by the Institutional Animal Care Research Advisory Committee of the National Institute of Biological Science and Animal Care Committee of Peking University Health Science Center. Animals were housed and allowed free access to tap water and maintained on a 12 h light/dark cycle. The ‘Principles of Laboratory Animal Care’ (NIH publication no. 85–23, revised 1996) were followed.

### Lipid analysis in plasma and liver

Blood was obtained by retro-orbital bleed after the mice were fasted for 16 h. Plasma total cholesterol (TC), TG and high density lipoprotein-cholesterol (HDL-C) were determined by using enzymatic methods (Sigma kits, MO, USA). Approximately 100 mg of liver was weighed and homogenized in 1 ml PBS. Lipids were extracted as described by Folchet al. [20] and dissolved in 100 ml 3 % Triton X-100. The determination of TG and TC was carried out using enzymatic methods as described previously. The liver free cholesterol (FC) and cholesteryl ester (CE) were determined by quantitation kit (BioVision, CA, USA).

### Plasma glucose, insulin, leptin, and adiponectin measurements

Plasma glucose were measured by enzymatic colorimetric methods using commercial kits (Sigma kits, MO, USA). For glucose tolerance test (GTT) and insulin tolerance test (ITT), mice were given glucose (2 g/kg body weight, i.p.; Abbott) or insulin (0.75 mIU/g body weight, i.p.; Humulin) after fasting for 4 h, respectively, and blood samples were collected before (time 0) and at 15, 30, 60 and 120 (90 for ITT) min after injection for measurement of glucose. Plasma insulin, leptin, and adiponectin levels were measured by ELISA (Linco Research, MO, USA).

### Histological studies

Liver tissue was fixed in 4 % neutral formalin. The paraffin-embedded sections were stained with hematoxylin/eosin (HE), and the cryostat sections thickness of 7 μm onto poly-l-lysine slides for lipid deposition analysis by oil red O (ORO) staining. The wild-type littermates were used as controls.

### RNA isolation and quantitative Real-time PCR

Total RNA was extracted using Trizol reagent (Invitrogen, USA) and first-strand cDNA was generated using RT kit (Invitrogen, USA). Amplifications were performed in 35 cycles using by using the Mx3000 Multiplex Quantitative PCR System (Stratagene, USA) with SYBR green fluorescence (Molecular Probes, Eugene, USA). Each cycle consisted of heating denaturation for 30 s at 94 °C, annealing for 30 s at 56 °C and extension for 30 s at 72 °C. All samples were quantitated by using the comparative CT method for relative quantitation of gene expression, normalized to glyceraldehyde-3-phosphate dehydrogenase (GAPDH) [21]. The primers used in this study were shown in Table 1. The results were represented by the ratio of the values to WT NC group.

**Table 1:** List of primers used in the experiment

Genes	Sense Primers	Antisense Primers
ACL	AATCCTGGCTAAAACCTCGCC	GCATAGATGCACACGTAGAACT
Acadm	AGGGTTTAGTTTTGAGTTGACGG	CCCCGCTTTTGTCATATTCCG
Acadl	TCTTTTCCTCGGAGCATGACA	GACCTCTCTACTCACTTCTCCAG
ACOX1	GGAGATCACGGGCACTTA	TGAGAATGAACTCTTGGGTC
AKT2	CAGATGGTCGCCAACAGT	TGCCGAGGAGTTTGAGATA
ChREBP	CCAGCCTCAAGGTGAGCAAA	CATGTCCCGCATCTGGTCA
CPT1α	TGGCATCATCACTGGTGTGTT	GTCTAGGGTCCGATTGATCTTTG
CPT2	CAGCACAGCATCGTACCCA	TCCCAATGCCGTTCTCAAAAT
DGAT1	ATCTGAGGTGCCATCGTC	ATGCCATACTTGATAAGGTTCT
Ehhadh	ATGGCTGAGTATCTGAGGCTG	GGTCCAAACTAGCTTTCTGGAG
Fas	GGTAGACAACAGCCGCATC	GTGTCCCATGTTGGATTTG
G6P1	AATCTCCTCTGGGTGGCA	GCTGTAGTAGTCGGTGTCC
IRS1	GGATCGTCAATAGCGTAA	GCTTGGCACAATGTAGAA
IRS2	GGGGCGAACTCTATGGGTA	GCAGGCGTGGTTAGGGAAT
MTP	GGAAAGCAGAGCGGAGAC	AGAGCAAGGGTCAGGCAC
PCK1	AGTCATCATCACCCAAGAGC	CCACCACATAGGGCGAGT
PGC-1α	CACAAACGATGACCCTC	GCATGTTGCGACTGC
PPAR-α	GGGCTTTCGGGATAGTTG	ATTGGGCTGTTGGCTGAT
PPAR-γ	GACCACTCGCATTCCTTT	CCACAGACTCGGCACTCA
SCD1	TGACCTGAAAGCCGAGAA	ATGTGCCAGCGGTACTCA
SREBP1c	TGGAGACATCGCAAACAAG	GGTAGACAACAGCCGCATC
GAPDH	TGATGACATCAAGAAGGTGGTGAAG	TCCTTGGAGGCCATGTAGGCCAT

### Western blot analysis

Mouse tissue was homogenized in RIPA assay buffer, and the protein content was determined using a bicinchoninic acid protein assay kit (Pierce, IL, USA). The following antibodies were used: apolipoprotein B (ApoB) and GAPDH (Millipore, MA, USA); AKT, phospho-AKT (Ser473) (Cell Signaling Technology, MA, USA); PPARα (GeneTex, CA, USA); SCD1 (BioVision, CA, USA); IRS1 (Santa Cruz Biotechnology, CA, USA); CPT1α (Proteintech, IL, USA). The protein bands were analyzed using densitometry and Image J image analysis software. The exam proteins levels were normalized to that of GAPDH. The results were represented by the ratio of the values to WT NC group.

### Statistical analysis

All data were presented as means ± SEM. Statistical comparisons between the four groups were performed using two-way ANOVA followed by Turkey test or followed by Mann–Whitney test for non-parametric data. A value of *P* < 0.05 was considered statistically significant.

## Results

### Effect of D/E diet on plasma lipid and ApoB protein levels in SKO mice

**Fig. 1:**
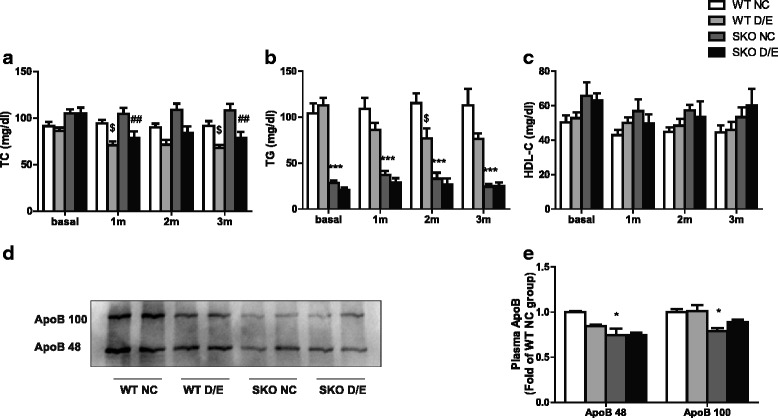
Plasma lipid and ApoB protein levels in WT and SKO mice with D/E diet. **a** plasma cholesterol levels (TC, *n* = 15-18); (**b**) plasma triglyceride levels (TG, *n* = 15-18); (**c**) high density lipoprotein levels (HDL-C, *n* = 10); (**d**) Western blot images for ApoB in plasma; (**e**) The quantification by densitometry of ApoB (*n* = 6). WT, wild type mice; SKO, seipin knockout mice; NC, normal chow diet;D/E, DHA/EPA 3:1 diet. Data are presented as mean ± SEM. *P < 0.05, ***P < 0.001, SKO NC group compared with WT NC group; ##*P* < 0.01, SKO D/E group compared with SKO NC group; $P <0.05, WT D/E group compared with WT NC group

As shown in Fig. 1a, plasma TC levels were reduced by D/E diet both in WT and SKO mice. Regarding plasma TG, although HTG is a common feature in human CGLs, it was not the same in the SKO mice. In fact, plasma TG levels in SKO mice were dramatically decreased by 70 % upon fasting (*P* < 0.001, as shown in Fig. 1b). Plasma TG levels decreased after 2 months with D/E diet in WT mice, but not changed in SKO mice (Fig. 1b). Plasma ApoB48 (264 kDa) and ApoB100 (520 kDa) levels were lower in SKO mice compared with WT mice (Fig. 1d, e). However, no difference was found for ApoB in both WT and SKO mice after D/E diet. Plasma HDL-C did not show any changes among the four groups (Fig. 1c).

### Improvement of D/E diet on insulin resistance in SKO mice

**Fig. 2:**
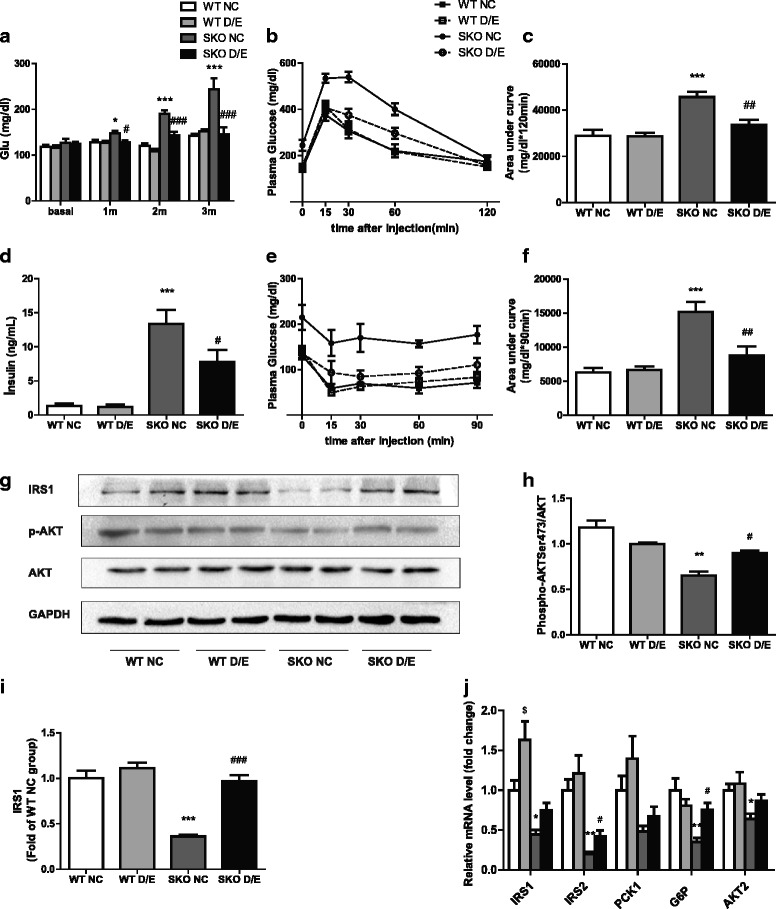
Glucose and insulin sensitivity related parameters. **a** Plasma glucose levels (Glu, *n* = 15-18); (**b**) Glucose tolerance test (GTT, *n* = 15-16); (**c**) Area under curve of glucose tolerance test; (**d**) serum insulin levels (*n* = 12); (**e**) Insulin tolerance test (ITT, *n* = 12); (**f**) Area under curve of insulin tolerance test; (**g**) Representative Western blot images for the proteins in liver; **h** The quantification by densitometry of phosphorylated AKT (p-AKT) normalized to total (*n* = 6); (**i**) The quantification by densitometry of IRS1 (*n* = 6); (**j**) Related gene expressions of insulin sensitivity in the liver (*n* = 12). WT, wild type mice; SKO, seipin knockout mice; NC, normal chow diet;D/E, DHA/EPA 3:1 diet. Data are presented as mean ± SEM. *P < 0.05, **P < 0.01, ***P < 0.001, SKO NC group compared with WT NC group; #*P* < 0.05, ##*P* < 0.01, ###*P* < 0.001, SKO D/E group compared with SKO NC group

We observed that the SKO mice displayed hyperglycemia after the age of 8 weeks compared with WT mice, and the hyperglycemia aggravated with the increase of age. D/E diet decreased plasma glucose in SKO mice, but was not in WT mice. This diet indeed revert the hyperglycemia of SKO mice to the similar levels in WT mice after 3 months of the treatment (Fig. 2a).

We found hyperinsulinemia, impaired glucose and insulin tolerance in SKO mice (Fig. 2b-f), as reported in previous study [13]. All of these abnormal parameters were improved dramatically by D/E diet (Fig. 2b-f). This indicated clearly that D/E diet ameliorated the hyperglycemia and insulin resistance caused by seipin deficiency.

To investigate the possible mechanisms, the mRNA and protein levels of insulin receptor substrates and glucose metabolism related genes such as IRS and AKT were examined in the livers. The gene expressions of IRS1, IRS2, glucose-6-phosohatase (G6P) and AKT2 was significantly reduced in SKO mice, however, D/E diet could reverse the down-regulation of IRS2 and G6P in SKO mice (Fig. 2j). The protein levels of IRS1 (136 kDa) and ratio of p-AKT /AKT (65 kDa) were reduced in SKO mice but up regulated by D/E diet (Fig. 2g–i).

### Improvement of hepatic lipid accumulation

**Fig. 3:**
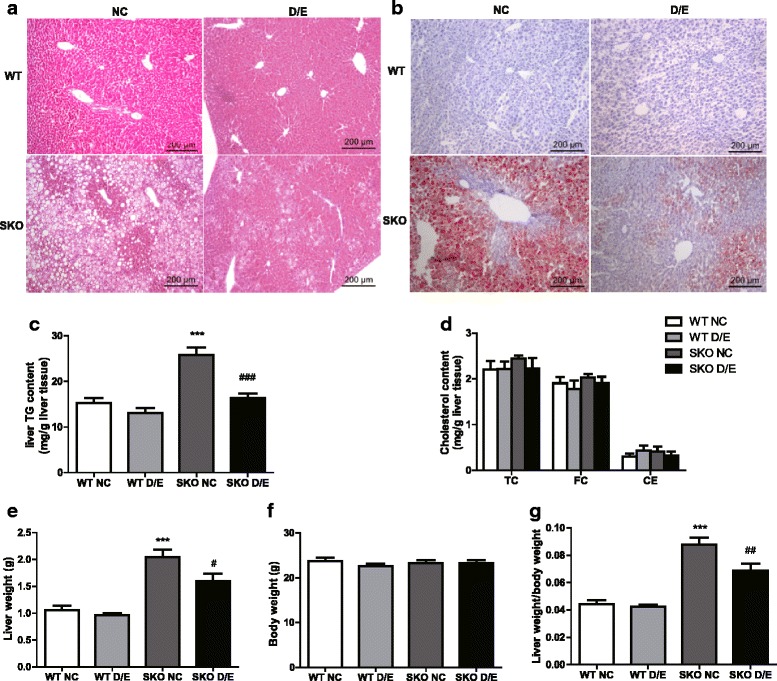
The improvement of hepatic lipid accumulation of SKO mice after D/E diet. **a** Histological analysis by hematoxylin/eosin staining for liver sections; (**b**) Representative photographs of the frozen hepatic sections by Oil Red O staining; (**c**) Triglyceride (TG) content in liver; (**d**) Hepatic Cholesterol (TC), free cholesterol (FC) and cholesteryl ester (CE) (*n* = 15-16); (**e**) Liver weight (*n* = 15-16); (**f**) Body weight (*n* = 15-16); (**g**) Liver weight/body weight (*n* = 15-16). WT, wild type mice; SKO, seipin knockout mice; NC, normal chow diet;D/E, DHA/EPA 3:1 diet. Data are presented as mean ± SEM. ***P < 0.001, SKO NC group compared with WT NC group; #*P* < 0.05, ##*P* < 0.01, ###*P* < 0.001, SKO D/E group compared with SKO NC group

Histologic analysis showed the SKO mice had severe hepatic steatosis by HE staining (Fig. 3a). There were fewer vesicles in SKO mice with D/E diet than NC diet. There were more lipid depositions in the livers of SKO mice by ORO staining but D/E diet could strongly reduce lipid accumulations in the livers (Fig. 3b).

We measured the content of TG in the livers of WT mice and SKO mice with NC or D/E diet respectively. The content of TG in SKO mice with NC diet was approximately doubled than that of WT mice before the D/E diet. After 3 months on D/E diet, it was decreased to the WT control levels (Fig. 3c). The accumulation of FC and CE in the livers had no difference among the four groups (Fig. 3d). The liver weight and ratio of the liver weight to body weight were increased in SKO mice with NC diet compared with WT mice, while D/E diet decreased this parameter by about 22 % (Fig. 3e–g), but did not change the content of TG, TC, FC or CE in WT mice.

### Changes of related gene expressions and protein levels of lipogenesis and β-oxidation in liver

**Fig. 4:**
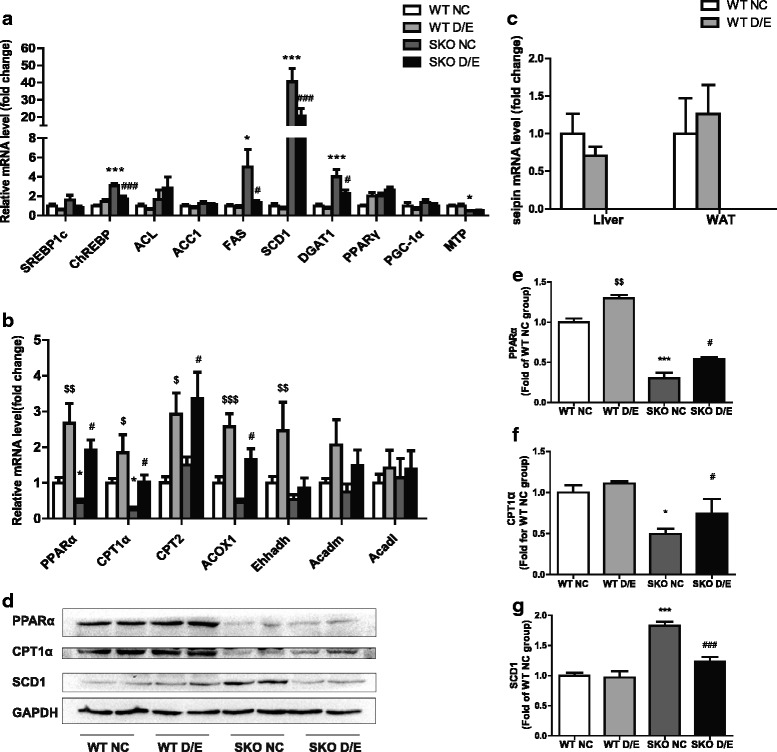
Gene expressions and protein levels in liver after 3 months of D/E diet. **a** Related gene expressions of lipogenesis in the liver (*n* = 12); (**b**) Related gene expressions of β-oxidation in the liver (*n* = 12); (**c**) seipin gene expression in liver and WAT (*n* = 12); (**d**) Representative Western blot images for the indicated proteins in liver; (**e**) The quantification by densitometry of PPARα (*n* = 6); (**f**) The quantification by densitometry of CPT1α (*n* = 6); (**g**) The quantification by densitometry of SCD1 (*n* = 6). WT, wild type mice; SKO, seipin knockout mice; NC, normal chow diet; D/E, DHA/EPA 3:1 diet. Data are presented as mean ± SEM. *P < 0.05, ***P < 0.001, SKO NC group compared with WT NC group; #*P* < 0.05, ###*P* < 0.001, SKO D/E group compared with SKO NC group; $P <0.05, $$P <0.01, $$$P <0.001, WT D/E group compared with WT NC group

De novo lipogenic gene transcripts including ChREBP, DGAT1, Fas and SCD1 were significantly increased in SKO mice compared with WT mice on NC diet, but significantly decreased after D/E diet (Fig. 4a). The protein level of SCD1 (37 kDa) showed the same tendency as the mRNA levels (Fig. 4d, g). It suggested that DHA/EPA could improve hepatic steatosis in the SKO mice through reducing the gene expressions of lipogenesis.

Hepatic β-oxidation related gene expressions were shown in Fig. 4b. The expressions of PPAR-α and CPT1α were lower in SKO than in WT mice. After diet with D/E, the expression of PPARα, CPT1α, CPT2 and ACOX1 were doubled in both WT and SKO groups. D/E diet could revert the decreases of PPARα and CPT1α gene expressions in SKO mice to the similar levels of WT mice on NC diet. PPARα and CPT1α protein were detected at 55 kDa and 88 kDa respectively and reduced in SKO mice. Again, diet with D/E could prevent these reductions (Fig. 4d-f).

We observed that the gene expression of microsomal transfer protein (MTP) was significantly down-regulated in SKO mice as described previously [13], and diet with D/E could not change it (Fig. 4a). Diet with D/E had also no effect on the gene expression of seipin in both liver and white adipose tissue (WAT) in WT mice (Fig. 4c).

### D/E diet had no significant effect on adipose tissue

**Fig. 5:**
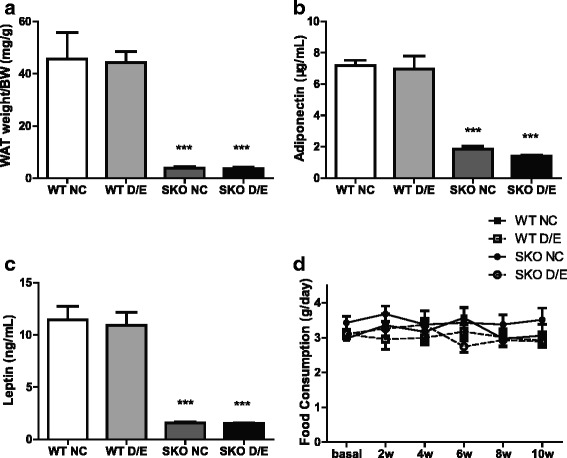
Food consumption and the changes in adipose tissue (*n* = 12). **a** Ratio of fat mass to body mass; (**b**) Plasma adiponectin levels; (**c**) Plasma leptin levels; (**d**) Food consumption at different time points. WT, wild type mice; SKO, seipin knockout mice; NC, normal chow diet; D/E, DHA/EPA 3:1 diet. Data are presented as mean ± SEM. ***P < 0.001, SKO group compared with WT group with the same diet

The fat mass in SKO mice was extremely less than that in WT mice, and diet with D/E had no effect on the ratio of WAT weight to body weight (BW) (Fig. 5a). The plasma adiponectin and leptin were significantly decreased in SKO mice, but no change after D/E diet (Fig. 5b, c). These data suggested that diet with D/E had no effect on adipose tissue in SKO mice. There was no statistically significant difference among the four groups in food consumption (Fig. 5d).

## Discussion

In this study, we found for the first time that diet rich in DHA/EPA could significantly ameliorate the hepatic steatosis caused by lipodystrophy in SKO mice. The hepatic TG content decreased about 40 % after DHA/EPA treatment for 3 months in these mice. These protective effects were probably due to the correction of the increased hepatic lipogenesis, insulin resistance and decreased β-oxidation in SKO mice by DHA/EPA.

Both obesity and lipodystrophy could induce hepatic steatosis in human. In this study, we found severe lipid deposition in the livers of both male and female SKO mice as previously reported [13]. In obesity, there were high expressions of hepatic SREBP-1c and ChREBP, which resulted in increased expression of hepatic lipogenic genes such as ACC, Fas and SCD1 [22]. These lead to hepatic de novo lipogenesis and steatosis in WT mice [23, 24]. However, in SKO lipodystrophy mice, only ChREBP but not SREBP1c was increased [13], which may affect the regulation of plasma glucose and insulin metabolism [25, 26]. This suggests that hepatic lipogenic regulations might be different between obesity and lipodystrophy. In this study, diet with DHA/EPA could reduce ChREBP gene expression. Thus the downstream genes Fas and SCD1 were also decreased, GTT and ITT were improved in SKO mice after the treatment [26]. These results were confirmed by the decreased SCD1 protein level (Fig. 4g). This may be one of the mechanisms that DHA/EPA improved the hepatic steatosis in SKO mice.

DHA/EPA has the beneficial effect on β-oxidation of the fatty acid in the liver which prevents NAFLD in high fat diet fed mice [7]. DHA/EPA could also improve defected β-oxidation in the liver of SKO mice. β-oxidation related genes such as PPARα and CPT1α were decreased both at mRNA and protein levels in SKO mice. It suggested a low level of β-oxidation in the liver of SKO mice [14, 27]. Diet with DHA/EPA could up-regulate the gene expressions of β-oxidation, such as PPARα, CPT1α, CPT2 and ACOX1 in both SKO and WT mice. The result indicates that diet with DHA/EPA could rescue the decreased β-oxidation in the livers of SKO mice to the same level with WT non treated mice, and this function may be one of the mechanisms of DHA/EPA in improving hepatic steatosis.

We found that SKO mice started to show hyperglycemia when they were 12 weeks old and hyperglycemia worsened with aging as described previously [13, 28]. The hyperinsulinemia, impaired GTT and ITT were also found in SKO mice. These results clearly indicated that SKO mice were insulin resistant [13]. After treatment of diet rich-in DHA/EPA for 3 months, hyperglycemia, hyperinsulinemia, impaired GTT and ITT were corrected. Insulin resistance plays an important role in hepatic steatosis, and the improvement of insulin sensitivity may be one of the mechanisms of DHA/EPA in correction of hepatic steatosis in SKO mice.

It has been shown that adipose tissue plays an important role in regulating lipid homeostasis by secretion of adipokines, like adiponectin and leptin, and is involved in hepatic steatosis and insulin resistance [29, 30]. Lopategi A et al. and Bjursell M et al. reported that DHA/EPA could improve hepatic steatosis through adipose tissue [30, 31]. Because adipose tissue is the major organ expressing seipin gene, it was then very important to know if DHA/EPA treatment could exert any effect on adipose tissue in SKO mice. Our data showed that fat mass/body weight ratio, plasma adiponectin and leptin were significantly decreased in SKO mice due to the lipodystrophy and there was no change in adipose tissue after diet with D/E. These data may indicated the beneficial effects of DHA/EPA to hepatic steatosis in SKO mice was independent of adipose tissue.

In this study, DHA/EPA could not reduce the plasma TG in SKO mice. However, several studies found that DHA/EPA could decrease plasma TG by inhibition of hepatic enzyme DGAT, which catalyzes the final reaction of TG synthesis. MTP is a molecular chaperone that catalyzes the rate-limiting step in very low-density lipoprotein assembly and secretion. A reduction of hepatic MTP gene expression and plasma ApoB48 level in SKO mice suggested that there might be a reduction of VLDL secretion [32]. This may explain the lack of hypertriglyceridemia in SKO mice and why DHA/EPA had no effect on TG levels in SKO mice. DHA/EPA also reduced the expression of DGAT1 in SKO mice. Likewise, VLDL may not be efficiently secreted from the liver in the absence of seipin, resulting in decreased plasma NEFA upon fasting [13]. We found that plasma cholesterols level decreased in both WT and SKO group after D/E diet. We hence tested the protein levels of ApoB48 and ApoB100 in plasma. They were lower in SKO mice than WT mice (Fig. 1d, e). The result of ApoB48 was confirmed by another study [32]. However, no difference was found for ApoB in both WT and SKO mice after D/E diet. This suggested the decrease of plasma TC was probably independent of the change of ApoB levels.

SKO mouse is one of congenital lipodystrophic animal models. Most other types of lipodystrophies may have the similar mechanisms for the development of hepatic steatosis, such as increased de novo lipogenesis, insulin resistance and reduced β-oxidation. Therefore, DHA/EPA diet might be also effective in other congenital lipodystrophic models or acquired lipodystrophy related to HIV-infected patients with antiretroviral treatment. Therefore, further investigations are thus warranted.

## Conclusions

Our data demonstrate that metabolic alterations by diet rich-in DHA/EPA in SKO mice could alleviate the hepatic steatosis in lipodystrophy. This function might be through reducing de novo lipogenesis,improving insulin resistance and increasing β-oxidation in the liver. Our findings suggest that the DHA/EPA diet might be a new effective treatment for disturbed metabolism in patients with lipodystrophy.

## Abbreviations

n-3 PUFAs: n-3 polyunsaturated fatty acids
EPA: Eicosapentaenoic acid
DHA: Docosahexaenoic acid
SKO: Seipin gene knockout
WT: Wild type
CGL: Congenital generalized lipodystrophy
NC: Normal chow diet
D/E: DHA/EPA diet
HDL-C: High density lipoprotein-cholesterol
Glu: Glucose
TC: Total cholesterol
TG: Triglycerides
GTT: Glucose tolerance test
ITT: Insulin tolerance test
ApoB: Apolipoprotein B
FC: Free cholesterol
CE: Cholesterol ester
ACL: ATP citrate lyase
Acadm: Medium-Chain fattyAcyl-CoA dehydrogenase
Acadl: Long Chain fatty Acyl-CoA dehydrogenase
ACOX1: Acyl-CoA Oxidase 1
AKT: Protein kinase B
ChREBP: Carbohydrate response element binding protein
CPT1α: Carnitine palmitoyltransferase-1α
CPT2: Carnitine palmitoyltransferase-2
DGAT1: Diacylgycerol acyltransferase 1
Ehhadh: Peroxisomal L-bifunctional enzyme
Fas: Fatty acid synthase
G6P: Glucose-6-phosphatase
IRS1: Insulin receptor substrate 1
IRS2: Insulin receptor substrate 2
MTP: Microsomal triglyceride transfer protein
PCK1: Phosphoenolpyruvate carboxykinase 1
PGC-1α: Peroxisome proliferator-activated receptor gamma, coactivator 1 alpha
PPARα: Peroxisome proliferator-activated receptor α
PPARγ: Peroxisome proliferator-activated receptor γ
SCD1: Stearoyl-CoA desaturase 1
SREBP1c: Sterol regulatory element binding protein-1c
GAPDH: Glyceraldehyde-3-phosphate dehydrogenase
